# Land cover and forest health indicator datasets for central India using very-high resolution satellite data

**DOI:** 10.1038/s41597-023-02634-w

**Published:** 2023-10-25

**Authors:** Sarika Khanwilkar, Chris Galletti, Pinki Mondal, Johannes Urpelainen, Harini Nagendra, Yadvendradev Jhala, Qamar Qureshi, Ruth DeFries

**Affiliations:** 1 Department of Ecology, Evolution, and Environmental Biology, Columbia University, New York, NY USA; 2 Department of Geography and Spatial Sciences, University of Delaware, Newark, DE USA; 3 Department of Plant and Soil Sciences, University of Delaware, Newark, DE USA; 4 Johns Hopkins School of Advanced International Studies, Maryland, DE USA; 5 School of Development, Azim Premji University, Bengaluru, India; 6 Wildlife Institute of India, Dehradun, India

**Keywords:** Forest ecology, Ecology

## Abstract

Satellite imagery has been used to provide global and regional estimates of forest cover. Despite increased availability and accessibility of satellite data, approaches for detecting forest degradation have been limited. We produce a very-high resolution 3-meter (m) land cover dataset and develop a normalized index, the Bare Ground Index (BGI), to detect and map exposed bare ground within forests at 90 m resolution in central India. Tree cover and bare ground was identified from Planet Labs Very High-Resolution satellite data using a Random Forest classifier, resulting in a thematic land cover map with 83.00% overall accuracy (95% confidence interval: 61.25%–90.29%). The BGI is a ratio of bare ground to tree cover and was derived by aggregating the land cover. Results from field data indicate that the BGI serves as a proxy for intensity of forest use although open areas occur naturally. The BGI is an indicator of forest health and a baseline to monitor future changes to a tropical dry forest landscape at an unprecedented spatial scale.

## Background & Summary

Forest cover changes impact global biodiversity and bio-geochemical cycles^1^ and livelihoods of forest-dependent people. Deforestation, the complete conversion of tree cover to another land cover, has been well-documented and quantified at regional and global scales using satellite imagery^2^. Technological developments in remote sensing methods have improved the feasibility to detect more fine-scale changes to forests; for example, Very-High Resolution (VHR) satellite data has increased the spatial resolution and amount of data available to make useful interpretations of land cover. Despite advancements in remote sensing, the scientific literature lacks a standard definition and methods for detecting and quantifying subtle ‘within class’ changes, such as forest degradation.

Generally, forest degradation is a change in the structure, function, or composition of a forest without complete loss of forest^3^. Soil health is included in different definitions of forest degradation because it is important for plant survival and growth. Additionally, lack of vegetation can lead to exposed soil (i.e. bare ground) within forests, which can alter soil moisture, water holding capacity, and nutrients^4^. Globally, the amount of exposed bare ground is increasing and from 2000 to 2012, an estimated 93,896 km^2^ of bare ground was gained^5^. The transition from tree cover to bare ground is caused by a complete loss of vegetation^6^, which may be due to resource extraction^5^.

The Central Indian Highlands Landscape (CIHL) spans across the Indian states of Madhya Pradesh, Maharashtra, and Chhattisgarh and is a heterogeneous mosaic of land covers that includes tree cover, exposed bare ground, water bodies, cropland, and villages and cities. The total geographic area is 273,136.6 km^2^. While there was only a slight decrease (1.7%) in total forest cover from 2003 to 2019 in the CIHL, there is evidence of nuanced changes to forest health; areas of open forest (canopy cover between 10% and 40%) and moderately dense forest (canopy cover between 40% and 70%), which made up a combined 83.0% of total forest in 2019, decreased by 4.9% and 7.5%, respectively, while very dense forest (canopy cover of 70% or more) increased by 30.5% (Supplementary Table 1)^7,8^.

Tropical Dry Forest (TDF) in the CIHL directly supports a high number of forest-dependent people (i.e. people living in and adjacent to forests and using the forest for livelihood needs and income generation), who largely belong to an officially recognized Scheduled Tribe or Scheduled Caste. Livestock rearing and agriculture are primary occupations. Livestock grazing and fire have altered tree species composition in the CIHL, which demonstrates the important long-term impacts associated with human use of the forest^9^. In addition, most forest-dependent households in the CIHL collect firewood for cooking fuel^10^. Another driver of forest degradation in the CIHL is lantana (*Lantana camara*), an invasive species which most often invades forests in India where humans lop trees for wood or graze livestock^11^.

In order to quantify and map forest health in the CIHL we first produce a high spatial resolution (3 meter (m)) land cover dataset. Several machine learning algorithms exist to classify land covers. We compared four machine learning algorithms based on an accuracy assessment and used the random forests (RF) algorithm^12^ to classify five land covers for the CIHL: tree cover, bare ground, water, cropland, and built environment. Tree cover was defined as woody vegetation excluding shrubs, bare ground was land without vegetation and lacking water (water) or man-made infrastructure (built environment), and cropland included fallow and active agriculture land. Based on the classification, we develop an index (Bare Ground Index, BGI) to quantify exposed bare ground within forested regions at 90 m. We assess the BGI with ground observations of signs of degradation, which include the presence of an invasive species as well as signs of resource extraction and forest use. Land cover and BGI datasets of central India are freely available in the GeoTIFF and KML file formats, respectively; code used to classify land cover and the BGI in Google Earth Engine are also available^13^. To our knowledge, this was the first VHR dataset of CIHL.

**Fig. 1:**
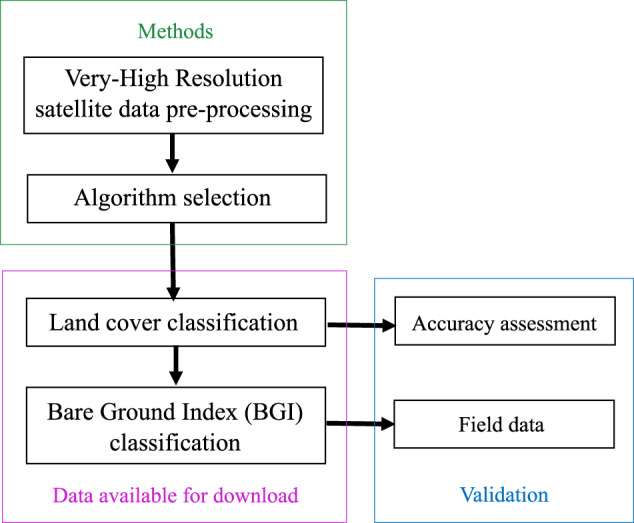
A flowchart outlining our approach to producing land cover and forest health indicator datasets in a Tropical Dry Forest using Very-High Resolution imagery.

The BGI is a structural indicator of forest health; it may be used as a baseline to monitor future changes to bare ground and tree cover in the CIHL and contribute towards an operational definition of forest degradation as one of several forest health indicators^14^. The BGI approach to understanding the status of forests in CIHL is distinct from previous efforts to map and quantify forests such as the Forest Survey of India because it integrates land cover with tree cover along with land cover without vegetation (bare ground). In addition, the BGI is derived from VHR data. Our approach (Fig. 1) to mapping the BGI can be applied to additional forested landscapes.

## Methods

### Very-high resolution (VHR) satellite data

Planet’s PlanetScope surface reflectance in 4 bands (Red, Green, Blue, and Near-infrared [NIR]) at 3 m resolution was used to classify land cover in the CIHL. The four spectral bands correspond to the following wavelengths: Red (590 to 670 nm), Green (500 to 590 nm), Blue (455 to 515 nm), and NIR (780 to 860 nm). We selected and downloaded images of the study area captured between February 28 and March 5 2018 using the Planet Explorer interface. Imagery during the winter season was selected to minimize cloud cover. Rainfall is highly seasonal and concentrated during the monsoon season (mid-June to September). Many tree species are deciduous and lose their leaves before the summer (March to mid-June). The coldest and driest season is from December to February. We aimed to capture bare ground exposed throughout the year because deciduous tree species maintain leaves in the winter. The images were mosaiced and clipped (i.e. pre-processed) into 233 tiles in ArcMap (Supplementary Fig. 1) and then uploaded into Google Earth Engine (GEE), which was the first step to testing algorithms, classifying land cover, and calculating the BGI.

### Algorithm selection

Four of the Planet imagery tiles, covering the fieldwork region were classified using RF, Support Vector Machine^15^, Boosted Decision Tree with AdaBoost, adaptive boosting^16^, and Kohonen’s Self Organizing Map with k-means clustering^17,18^.

Random Forest is an ensemble classification algorithm based on a collection of decision trees; the starting node, or root of the tree, considers all training data. The first and subsequent splits separate the training data into subsets by using the input features (image bands). Support Vector Machine is a non-parametric classifier that creates a linear decision boundary for a dataset based on support vectors, a subset of the training samples. AdaBoost, short for adaptive boosting, is an ensemble method that sequentially combines the results of weak estimators, such as individual decision trees, to obtain an optimal classification^16^. Finally, Kohonen’s Self Organizing Map with k-means clustering is an unsupervised neural network that uses competitive learning to optimize a vector of weights, or “synaptic coefficients,” of a given set of neurons to minimize the distance between each input vector and its associated neuron^17,18^.

We assessed the performance of each ML algorithm based on the overall accuracy and the kappa index, and selected RF as the best performing algorithm (Table 1). A total of 18 models were run which differed in the algorithm used and the number of samples in the training data (Supplementary Table 2) and algorithm parameters (Supplementary Tables 3–6). The final accuracy of all models was assessed using validation data from a stratified random sample of pixels which were distributed across the four test tiles. The randomization was stratified by class and by geography. There were 5,332 total pixels assessed with a minimum of 150 pixels per class. For geographic stratification, a uniform grid was established across the corridor and pixels were randomly spread across the cells within the grid.

**Table 1:** Algorithm selection was accomplished by comparing the performance of four machine learning (ML) algorithms in the land cover classification of the fieldwork region of the Central Indian. Highlands Landscape. Four Planet tiles that were also used to produce the final landscape classification were classified and the Random Forest ML algorithm resulted in the highest overall accuracy and kappa index (indicated by a *).

Classification type	Algorithm	Highest overall accuracy	Kappa
Supervised	Random Forest	0.70*	0.61*
	Support Vector Machine	0.44	0.32
	Boosted Decision Tree (AdaBoost)	0.69	0.60
Unsupervised	Kohonen’s Self Organizing Map	0.63	0.51

Training data for each land cover class was selected as polygons using Google Earth imagery from February 2018. Pixels within the polygons were extracted and assigned a land cover class. The same training data was used to train all three supervised algorithms. Likewise, the same validation was used to assess the accuracy of each algorithms’ classification output. Models were trained using the Scikit-Learn package within Python v2.7 and parameters varied. The ML models were then applied to the images on a Linux-based high-performance computing cluster that processed each image in just over an hour.

### Land cover classification

**Fig. 2:**
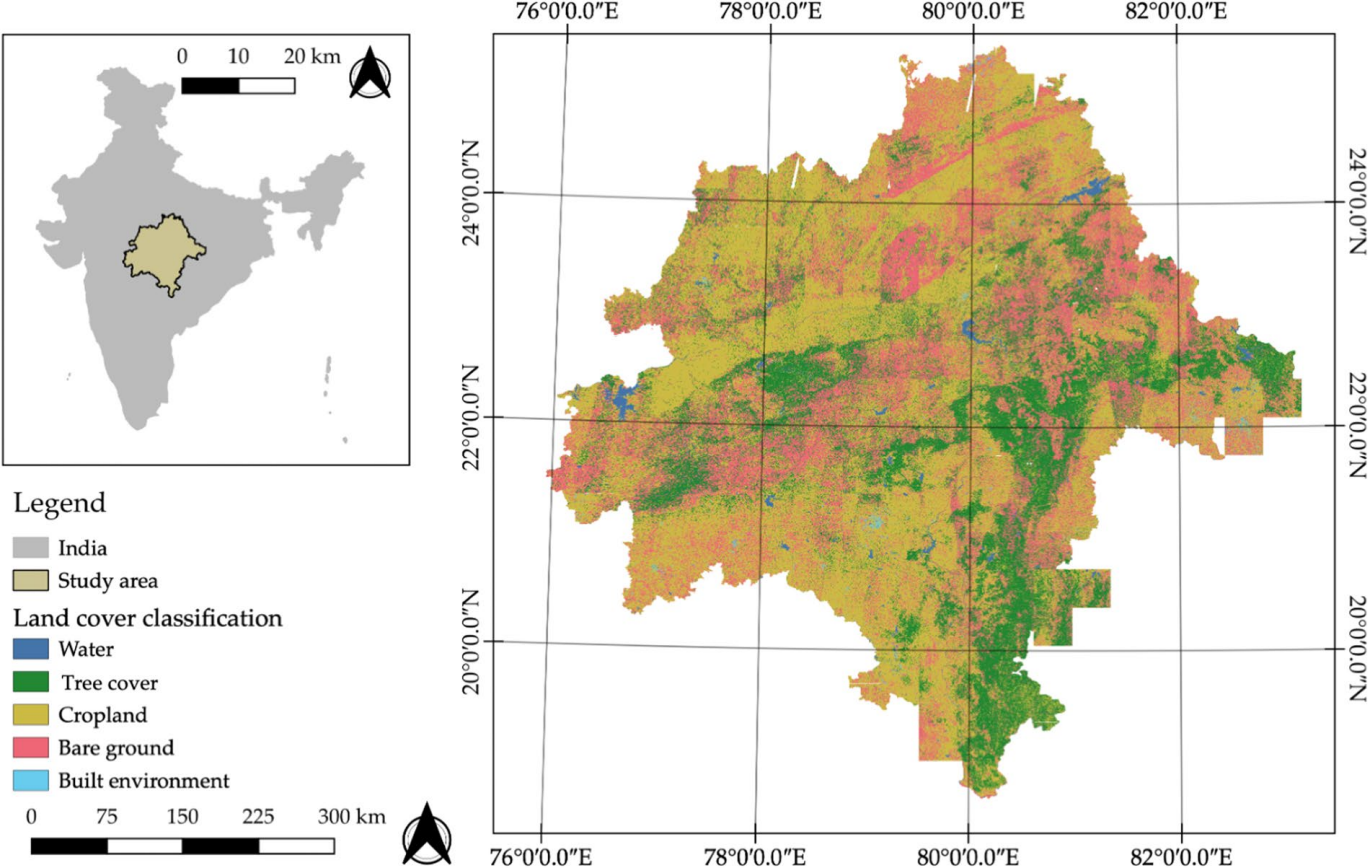
Very-High Resolution (3 meter) land cover map of the Central Indian Highlands Landscape. The classification was completed in Google Earth Engine and visualized in QGIS3.16.

Each Planet tile was classified into five discrete land covers: trees, bare ground, water, cropland, and built environment (Fig. 2). Data is available in GeoTIFF format, with coordinate reference system (CRS) WGS 84 (EPSG: 32643 or 32644) (Supplementary Fig. 1), and includes 3 m resolution land cover information (class 1: water, class 2: tree, class 3 and 5: cropland, class 4: bare ground, class 6: built environment). Cropland represents an aggregation of fallow and active cropland. We identified trees and bare ground in order to derive the BGI and additional land cover types were chosen based on a field assessment completed within the fieldwork region in February 2018. Training data for each land cover class from the algorithm selection was used, and additional training data was selected as polygons using Google Earth imagery from February 2018 and collected in the fieldwork region in February 2018 and June 2019. Pixels, 1,048,575 in total, within the polygons were extracted and assigned a land cover class (Table 2). The pixels were used as training data using a RF classifier, with 10 decision trees as a parameter in the RF classifier, in Google Earth Engine’s Classifier package and was applied to Planet imagery at 3 m scale in GEE.

**Table 2:** The mean and standard deviation (SD) of reflectance values of all the training data according to land cover type.

Band	Tree cover, N = 498,049	Bare ground, N = 130,756	Cropland, N = 95,864	Water, N = 215,989	Built environment, N = 107,917
Red, Band 1 Mean (SD)	437.67 (52.21)	1077.70 (215.90)	476.97 (75.17)	552.80 (70.18)	824.59 (130.24)
Green, Band 2 Mean (SD)	544.14 (62.57)	1312.09 (258.84)	603.97 (82.94)	673.54 (100.03)	996.97 (157.97)
Blue, Band 3 Mean (SD)	580.90 (85.51)	1733.98 (378.31)	574.24 (133.72)	675.71 (112.33)	1236.04 (225.10)
Near-infrared, Band 4 Mean (SD)	1967.48 (265.02)	2828.61 (438.59)	3336.76 (756.40)	703.57 (127.33)	2171.17 (368.07)

### Bare ground index (BGI) classification

**Fig. 3:**
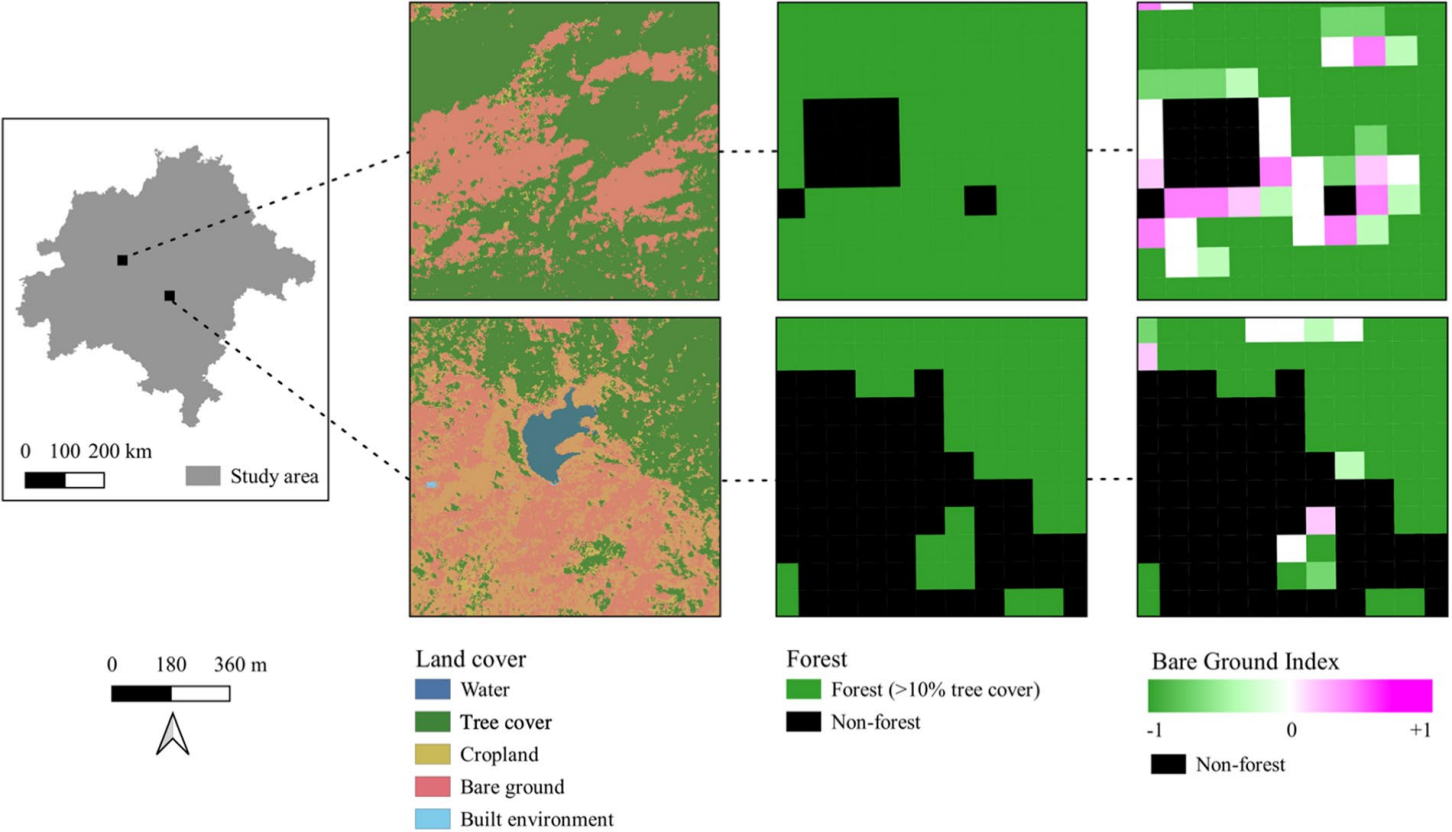
The Bare Ground Index (BGI) was calculated and mapped in the Central Indian Highlands Landscape at 90 meter (m) resolution. The BGI was derived from the land cover classification. First, forest (>10% tree cover within a 90 m pixel) and non-forest was identified. Then, the BGI, a normalized index that ranges from −1 to +0.8, was identified within forest. White indicates pixels where the BGI equals 0. Pixels that are pink have more bare ground as compared to tree cover, whereas pixels that are green have more tree cover as compared to bare ground.

The BGI (Fig. 3) was calculated and mapped using land cover data from the VHR land cover classification. First, we aggregated the land cover to 90 m resolution to identify forest, defined as >10% tree cover^19^, and non-forest (<10% tree cover). Then, we calculated the BGI within forest.

The BGI is a normalized index that ranges from −1.0 (all forest) to +0.8 (all bare ground). The maximum BGI value for a pixel is +0.8 because the BGI was only calculated within pixels that had 10% or greater tree cover. The BGI was derived from the land cover classification and calculated using the following equation:

1 $${\rm{Bare}}\;{\rm{Ground}}\;{\rm{Index}}({\rm{BGI}})=({{\rm{BareGround}}}_{{\rm{i}}}-\,{{\rm{TreeCover}}}_{{\rm{i}}})/({{\rm{BareGround}}}_{{\rm{i}}}+{{\rm{TreeCover}}}_{{\rm{i}}})$$

where BGI is the Bare Ground Index (BGI) at 90 m resolution, and TreeCover_i_ and BareGround_i_ is the fraction of pixels within the 90 m pixel that were classified as “tree cover” and “bare ground,” respectively, in the land cover classification. The BGI classification was performed using the GEE Code Editor (www.code.earthengine.google.com) and visualized in QGIS3.16. Figure 3 shows examples of the BGI. The BGI classification is available in KML format at CRS WGS 84 (EPSG: 4326) and includes attributes such as the BGI and fraction of pixels classified as bare ground and tree cover (Supplementary Table 7).

## Data Records

Data are available for download from Science Data Bank at 10.57760/sciencedb.10422^13^. The ‘Read_me’ PDF file describes the available land cover and BGI classification data files. The ‘code’ Word file includes the code used in the GEE Code Editor for the land cover and BGI classifications. The ‘StudyAreaZones’ shapefile shows the location of 233 tiles that cover the landscape; the tile number corresponds to the location and file name of available land cover (‘Classified_[tile number]’) and BGI (‘classified_bgi_[tile number]’) data.

## Technical Validation

### Land cover classification accuracy

In addition to assessing the accuracy of multiple ML algorithms during algorithm selection, we conducted an accuracy assessment of the final land cover dataset following an independent resampling approach (Table 3). Geographic randomization of reference data was achieved by generating ten random points per tile (2,330 points) in R version 3.6 to ensure an unbiased reference data selection and distribution across the study area. Reference data was selected through visual interpretation of historical imagery in Google Earth Pro from the same season and year as Planet imagery by manually delineating homogeneous land cover polygons around the ten randomly selected points. When historical imagery was not available, land cover from the closest year prior to 2018 was interpreted. If imagery prior to 2018 was interpreted and land cover had changed between that time and the present at the randomly selected point, reference data was selected from a location nearest the random point and where land cover class remained between the historical data and post-2018 imagery. The accuracy of the land cover classification as compared to our reference data was calculated in R version 3.6 and resulted in 83.00% overall accuracy (95% confidence interval (CI): 61.25%–90.29%). The 95% CI for overall accuracy was calculated using the mean and standard error of user and producer accuracies (Eq. 2, Table 3). The user accuracy for tree and bare ground classes were 90.21% (95% CI: 89.87%–90.54%) and 52.19% (95% CI: 51.55%–52.83%), respectively; the producer accuracy for tree and bare ground classes were 88.53% (95% CI: 88.16%–88.88%) and 92.08% (95% CI: 91.61%–92.53%), respectively (Table 3). The 95% CI for each user and producer accuracy was calculated using Eq. 3, where *x* is the number of positive samples and *N* is the number of total samples (Table 3).

2 $$Overall\;accuracy\,95{\rm{ \% }}\;CI=Mean\pm 1.96\times \frac{Standard\;Deviation}{\sqrt{10}}$$

3 $$User\;or\;producer\;accuracy\,95{\rm{ \% }}\,CI=\frac{x}{N}\pm 1.96\times \sqrt{x\times \frac{N-x}{{N}^{3}}}$$

**Table 3:** Error matrix to assess the accuracy of the final land cover dataset. Reference data were polygons of a single land cover identified from 10 randomized points per tile; land cover was identified from historical Google Earth imagery. The overall accuracy was 83.00% (95% Confidence Interval (CI): 61.25%–90.29%).

Land cover classification	Independent samples					Row total	User’s accuracy (%)	User’s accuracy 95% CI (lower limit, upper limit)
	Tree cover	Bare ground	Built environment	Cropland	Water			
**Tree cover**	**27220**	17	56	2625	256	30174	90.21	89.87, 90.55
**Bare ground**	1357	**12252**	156	4716	4995	23476	52.19	51.55, 52.83
**Built environment**	1	208	**2623**	2655	7870	13357	19.64	18.96, 20.31
**Cropland**	1111	827	569	**39984**	9274	51765	77.24	76.88, 77.60
**Water**	1059	2	7	47	**102446**	103561	98.92	98.86, 98.99
Column total	30748	13306	3411	50027	124841	222333		
Producer’s accuracy (%)	88.53	92.08	76.90	79.92	82.06			
Producer’s accuracy 95% CI (lower limit, upper limit)	88.17, 88.88	91.62, 92.54	75.48, 78.31	79.57, 80.28	81.85, 82.27			

### Ground validation of the BGI

**Fig. 4:**
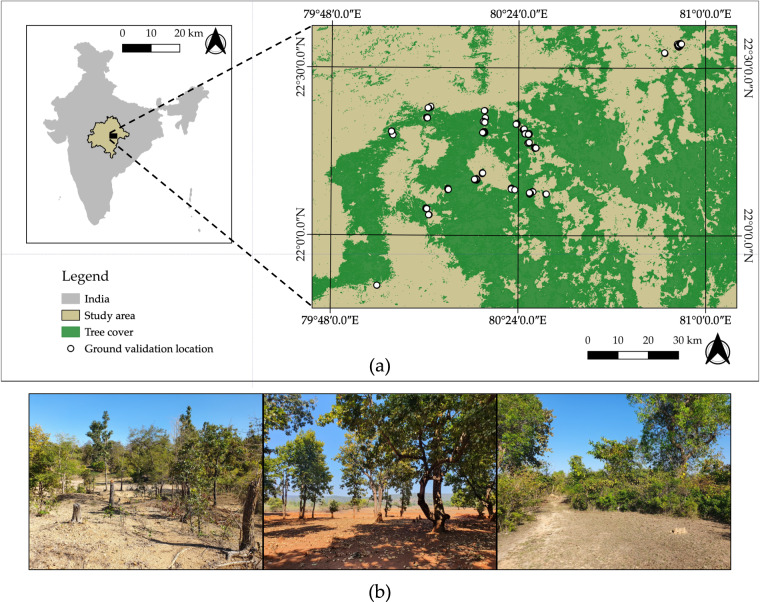
Ground validation data was collected from the fieldwork region of the study area in February 2020 (**a**). Tree cover comes from Hansen *et al*.^2^. Trails were present in photos from ground validation locations (**b**) that illustrate exposed bare ground within forests which we aimed to identify and map with the Bare Ground Index (BGI). Signs of lopping were present in the far-left photo. Photos were taken with a Samsung Galaxy S10+.

In February 2020, we visited 191 locations which varied from high to low BGI in the fieldwork region (Fig. 4). The season of data collection during ground validation coincided with the season that Planet satellite images were acquired, which was of particular importance to accommodate the seasonality of the region. Signs of forest use and invasive species presence, including trails, cattle dung, and lopping, and lantana were detected within a 10 m radius of each ground validation location. These signs were visually assessed into 3 categories: (1) no sign of forest use or lantana plant, (2) 1 or 2 trees lopped, dung piles, trails, or lantana plants, and (3) 3 or more trees lopped, dung piles, trails, or lantana plants. The visual assessment was quantified as 0 (no signs), 1 (1 or 2 signs), or 2 (3 or greater signs). Then, we compared the BGI values of areas with minimal to maximal signs of forest use and lantana using a Wilcoxon rank sum test, which estimates the significance of the difference between non-normally distributed data. There were significantly higher amounts (p < 0.05) of cattle dung in places with higher values of BGI, or more bare ground than tree cover (Supplementary Fig. 2). There were no significant associations between the BGI and other signs of forest use.

## Usage Notes

There is not a single remote sensing method that can measure forest health and degradation in all of its complexity. Where changes to the forest cannot be measured through changes in tree cover alone, the BGI serves as a geospatial tool to quantify and explore one characteristic of forest health. The BGI can be a valuable metric to couple with other indicators of forest health to assess and understand forest degradation and contributes to a broader need to assess and estimate changes to forest health in TDFs around the world, forests which have been understudied as compared to tropical moist or wet forests^20^.

An increase in BGI is associated with increased presence of cattle dung, which we used as a proxy for intensity of cattle grazing. Stronger evidence that links the BGI directly to cattle grazing can be collected through further field research, such as direct observations of cattle grazing and a more thorough understanding of grazing patterns through social surveys. Throughout our ground surveys of the BGI, signs of forest use were prevalent across a range of values of the BGI. Such activities may continue to impact forest health below the canopy where optical data is unable to detect. We advocate for the development of additional forest heath indicators using LiDAR and SAR data, with a specific emphasis on identifying indicators of degraded forest structure and composition driven by lantana invasion, firewood collection, and human and livestock movement through the forest.

Phenological and historical examinations of the BGI would provide further insight into structural changes to the forest. We carefully considered the dates of image acquisition and ground validation due to seasonality of vegetation (see Very-High Resolution (VHR) satellite data section). Although exposed bare ground occurs naturally in some locations in the study area as well of other TDFs, we measured tree cover during a season when a majority of the deciduous tree species had leaves. Historical VHR data may be used to detect long term persistence of, or changes to, the BGI. For example, transitions from tree cover to bare ground which would be indicated by increased BGI values. Future users of the BGI data and/or methods must consider inter and intra annual vegetation cycles before making interpretations and comparisons of the BGI.

It is not possible to compare the BGI of a forest across large geographies where forest types and vegetation differ. The BGI we produced was derived from five land cover classes; forms of vegetation such as shrubs or grasses were included in one of the classified land covers. For instance, lantana may have been classified under the tree cover class because we found greater amounts of lantana in areas with low BGI values compared to high BGI values and this difference approached significance (p = 0.07) (Figure S1). Grasses were likely classified in cropland because both can be seasonal land covers or have similar vegetation structures, although there was no technical validation to quantify the error. Bare ground was commonly misclassified as cropland or water, and built environment was largely misclassified as water.

We advocate that others adapt our methods to monitor the BGI in additional TDFs and derive the BGI from land cover classification with a larger number of vegetation classes. Deriving the BGI from a more distinctive tree cover class could help overcome potential issues of interpretation similar to the Normalized Difference Vegetation Index (NDVI), a measure of live vegetation cover, where the NDVI value is not limited to photosynthetic activity from trees alone^21^. Finally, additional indicators of forest health in the CIHL can be developed that incorporate locally grounded values, knowledges, and needs^22^.

### Supplementary information

Supplementary Information

## Data Availability

The code classifying land cover from PlanetScope imagery and deriving the BGI was written in Google Earth Engine. The JavaScript language to classify land covers from Planetscope imagery and derive the BGI from the land cover is available as the ‘Code’ text file from Science Data Bank at 10.57760/sciencedb.10422/.
