# Supplementary material for: Land cover and forest health indicator datasets for central India using very-high resolution satellite data
Source: Sci Data. 2023 Oct 25;10:738. doi: 10.1038/s41597-023-02634-w (PMC10600235; doi:10.1038/s41597-023-02634-w)
Supplement: Supplementary file 1 — Supplementary Information [file 41597_2023_2634_MOESM1_ESM.docx]

**Supplementary Materials**

**Table of contents**

Page #

1. Supplementary Table 1…………………………………………………………………….2

2. Supplementary Table 2…………………………………………………………………….4

3. Supplementary Table 3…………………………………………………………………….5

4. Supplementary Table 4…………………………………………………………………….6

5. Supplementary Table 5…………………………………………………………………….7

6. Supplementary Table 6…………………………………………………………………….8

7. Supplementary Table 7…………………………………………………………………….9

8. Supplementary Figure 1……………………………………………………………………10

9. Supplementary Figure 2……………………………………………………………………11

**Supplementary Table 1.** There were 38 districts in the study area (Central Indian Highlands Landscape). We report on total forest cover, open forest (land with tree cover of canopy density between 10% and 40%), moderately dense forest (land with tree cover of canopy density between 40% and 70%), and very dense forest (land with tree cover of canopy density above 70%) in 2003 and 2019 for 37 districts. Madhya Pradesh’s Annapur district had no data in 2003 so we do not include forest cover data from this district. Source: Forest Survey of India, 2003^7^ and 2019^8^.

| **State** | **District** | **Total**  **Forest**  **Cover**  **2003** | **Open**  **Forest**  **2003** | **Moderately**  **Dense**  **Forest**  **2003** | **Very**  **Dense**  **Forest**  **2003** | **Total**  **Forest**  **Cover**  **2019** | **Open**  **Forest**  **2019** | **Moderately**  **Dense**  **Forest**  **2019** | **Very**  **Dense**  **Forest**  **2019** |
| --- | --- | --- | --- | --- | --- | --- | --- | --- | --- |
| Madhya Pradesh | Chhatarpur | 1706 | 862 | 803 | 41 | 1758.55 | 756.97 | 817.52 | 184.06 |
| Madhya Pradesh | Panna | 2728 | 1069 | 1595 | 64 | 2742.71 | 1181.44 | 1478.26 | 83.01 |
| Madhya Pradesh | Sagar | 2922 | 1198 | 1722 | 2 | 2794.54 | 1651.97 | 1141.57 | 1 |
| Madhya Pradesh | Damoh | 2678 | 1769 | 903 | 6 | 2587.18 | 1739.39 | 845.79 | 2 |
| Madhya Pradesh | Satna | 1678 | 717 | 942 | 19 | 1752.9 | 831.2 | 909.7 | 12 |
| Madhya Pradesh | Rewa | 708 | 474 | 224 | 10 | 781.15 | 333.57 | 386.58 | 61 |
| Madhya Pradesh | Umaria | 1872 | 528 | 1108 | 236 | 2022.58 | 548.05 | 1096.22 | 378.31 |
| Madhya Pradesh | Vidisha | 902 | 375 | 495 | 32 | 777.46 | 431.55 | 344.91 | 1 |
| Madhya Pradesh | Bhopal | 312 | 215 | 97 | 0 | 328.67 | 207.75 | 120.92 | 0 |
| Madhya Pradesh | Sehore | 1464 | 724 | 740 | 0 | 1357.9 | 719.15 | 614.85 | 23.9 |
| Madhya Pradesh | Raisen | 2732 | 1084 | 1569 | 79 | 2676.26 | 1346.75 | 1306.51 | 23 |
| Madhya Pradesh | Betul | 3537 | 1551 | 1844 | 142 | 3663.7 | 1495.22 | 1938.14 | 230.34 |
| Madhya Pradesh | Harda | 1045 | 446 | 598 | 1 | 956.26 | 409.57 | 527.69 | 19 |
| Madhya Pradesh | Hoshangabad | 2402 | 849 | 1292 | 262 | 2422.65 | 780.44 | 1370.32 | 271.89 |
| Madhya Pradesh | Katni | 1191 | 625 | 477 | 89 | 1361.3 | 658.82 | 608.58 | 93.9 |
| Madhya Pradesh | Jabalpur | 1078 | 620 | 408 | 50 | 1113.93 | 570.43 | 502.5 | 41 |
| Madhya Pradesh | Narsimhapur | 1374 | 783 | 517 | 74 | 1342.76 | 624.42 | 657.34 | 61 |
| Madhya Pradesh | Dindori | 2643 | 592 | 1478 | 573 | 3031.96 | 663.85 | 1281.17 | 1086.94 |
| Madhya Pradesh | Mandla | 2732 | 980 | 1309 | 443 | 2577.51 | 795.15 | 1091.05 | 691.31 |
| Madhya Pradesh | Chhindwara | 4409 | 1838 | 2368 | 203 | 4588.01 | 1938.98 | 2027.09 | 576.94 |
| Madhya Pradesh | Seoni | 3038 | 1387 | 1412 | 239 | 3069.59 | 1041.37 | 1791.14 | 237.08 |
| Madhya Pradesh | Balaghat | 4859 | 1682 | 2547 | 630 | 4932.06 | 883.84 | 2638.97 | 1409.25 |
| Madhya Pradesh | Shahdol | 2483 | 893 | 1491 | 99 | 1970.71 | 1028.17 | 820.54 | 122 |
| Madhya Pradesh | East Nimar | 3580 | 1479 | 2058 | 43 | 2089.12 | 784.52 | 1156.8 | 147.8 |
| STATE TOTAL |  | 54073 | 22740 | 27997 | 3337 | 52699.46 | 21422.57 | 25474.16 | 5757.73 |
| Maharashtra | Akola | 321 | 195 | 111 | 15 | 340.37 | 220.93 | 108.44 | 11 |
| Maharashtra | Amravati | 3069 | 997 | 1395 | 677 | 3167.77 | 1087.35 | 1461.53 | 618.89 |
| Maharashtra | Wardha | 824 | 386 | 438 | 0 | 861.95 | 441.95 | 410.03 | 9.97 |
| Maharashtra | Nagpur | 1984 | 664 | 961 | 359 | 2000.38 | 696.76 | 902.56 | 401.06 |
| Maharashtra | Bhandara | 886 | 223 | 526 | 137 | 998.92 | 264.93 | 563.13 | 170.86 |
| Maharashtra | Gondiya | 2160 | 461 | 887 | 812 | 1938.59 | 317.75 | 732.23 | 888.61 |
| Maharashtra | Gadchiroli | 10069 | 2143 | 3725 | 4201 | 9916.94 | 1909.92 | 3307.73 | 4699.29 |
| Maharashtra | Chandrapur | 3940 | 1039 | 1639 | 1262 | 4054.46 | 1171.99 | 1559.44 | 1323.03 |
| STATE TOTAL |  | 23253 | 6108 | 9682 | 7463 | 23279.38 | 6111.58 | 9045.09 | 8122.71 |
| Chhattisgarh | Korba | 3358 | 1023 | 2186 | 149 | 3393.7 | 877.08 | 2313.62 | 203 |
| Chhattisgarh | Janfgir-Champa | 157 | 102 | 51 | 4 | 149.89 | 125.76 | 22.13 | 2 |
| Chhattisgarh | Bilaspur | 2504 | 600 | 1682 | 222 | 2456.89 | 522.7 | 1539.19 | 395 |
| Chhattisgarh | Kabeerdham | 1621 | 375 | 1246 | 0 | 1548.72 | 385.79 | 1083.84 | 79.09 |
| Chhattisgarh | Rajnandgaon | 2548 | 818 | 1727 | 3 | 2535.18 | 754.67 | 1749.51 | 31 |
| STATE TOTAL |  | 10188 | 2918 | 6892 | 378 | 10084.38 | 2666 | 6708.29 | 710.09 |
| **STUDY AREA TOTAL** |  | **87514** | **31766** | **44571** | **11178** | **86063.22** | **30200.15** | **41227.54** | **14590.53** |

**Supplementary Table 2**. Overall accuracy and kappa index for a total of 18 models which were run using Random Forest, Support Vector Machine, Boosted Decision Tree (AdaBoost), or Kohonen’s Self Organizing Map with k-means clustering. Models differed in the algorithm used, the number of samples in the training data, and algorithm parameters. Algorithm parameters are specific in Table S3. For each algorithm, the highest overall accuracy and kappa index is in bold, these four models are reported in Table 1 (in-text). * denotes the model with highest overall accuracy and kappa index, run using the Random Forest algorithm.

| **Algorithm** | **# of samples in training data** | **Model** | **Overall Accuracy** | **Kappa** |
| --- | --- | --- | --- | --- |
| Random Forest | 6,000 pixels | 1 | 0.694 | 0.606 |
|  |  | 2 | 0.692 | 0.606 |
|  |  | 3 | 0.693 | 0.605 |
|  | 18,000 pixels | 4 | **0.697*** | **0.610*** |
|  |  | 5 | 0.695 | 0.608 |
|  |  | 6 | 0.696 | 0.609 |
|  | Polygons | 7 | 0.693 | 0.602 |
|  |  | 8 | 0.690 | 0.598 |
|  |  | 9 | 0.696 | 0.606 |
| Support Vector Machine | 6,000 pixels | 10 | 0.378 | 0.267 |
|  |  | 11 | 0.427 | 0.315 |
|  | 18,000 pixels | 12 | 0.388 | 0.279 |
|  |  | 13 | **0.435** | **0.324** |
| Boosted Decision Tree (AdaBoost) | 6,000 pixels | 14 | **0.687** | **0.597** |
|  | 18,000 pixels | 15 | 0.680 | 0.588 |
| Kohonen’s Self Organizing Map | - | 16 | 0.622 | 0.507 |
|  |  | 17 | **0.631** | **0.514** |
|  |  | 18 | 0.556 | 0.416 |

**Supplementary Table 3**. The name of model parameters that were varied. Specific model parameters are listed in Table S4 to S6.

| **Classification Type** | **Model Name** | **Algorithm** | **Parameters** |
| --- | --- | --- | --- |
| Supervised Classifications | 1 | Random Forest | Number of trees (n_estimators) |
|  | 2 |  |  |
|  | 3 |  |  |
|  | 4 |  |  |
|  | 5 |  |  |
|  | 6 |  |  |
|  | 7 |  |  |
|  | 8 |  |  |
|  | 9 |  |  |
|  | 10 | Support Vector Machine | RBF kernel: C, error rate |
|  | 11 |  |  |
|  | 12 |  | Polynomial kernel: C, degree |
|  | 13 |  |  |
|  | 14 | Boosted Decision Tree (Adaboost) | n_estimators, learning rate |
|  | 15 |  |  |
| Unsupervised Classifications | 16 | Kohonen's Self Organizing Map with k-means clustering | Number of output neurons |
|  | 17 |  |  |
|  | 18 |  |  |

**Supplementary Table 4**. Model parameters for models 1 to 0 (run using Random Forest algorithm).

| **Model** | **Number of trees** |
| --- | --- |
| 1, 4, and 7 | 50 |
| 2, 5, and 8 | 100 |
| 3, 6, and 9 | 200 |

**Supplementary Table 5**. Model parameters for models 10 to 13 (run using the Support Vector Machine algorithm).

| **Model** | **C** | **gamma** | **Degree** |
| --- | --- | --- | --- |
| 10 and 12 | 800 | 0.90 | - |
| 11 and 13 | 1000 | - | 1 |

**Supplementary Table 6**. Model parameters and quantization error for models 16, 17, and 18 (run using the Kohonen’s Self Organizing Map algorithm).

| **Parameters** | **Model #** | | |
| --- | --- | --- | --- |
|  | **16** | **17** | **18** |
| Output layer neurons: | 100 | 225 | 400 |
| Min learning rate: | 0.5 | | |
| Max learning rate: | 1 | | |
| k-means clustering: | yes | | |
| Max no. of output clusters: | 32 | | |
| Quantization error: | 0.0197 | 0.017 | 0.015 |
| Iterations: | 12,715,912 | | |

**Supplementary Table 7.** Description of attributes in the BGI classification data.

| **Column name** | **Explanation** |
| --- | --- |
| BGI | The BGI value, ranging from -1.0 to +0.8. Non-forest areas where the BGI was not calculated will have a BGI value of -10000 |
| bareground_percent | Fraction of pixels classified as bare ground |
| tree_percent | Fraction of pixels classified as tree cover |
| histogram | List of histogram of classes within 90 m pixel to calculate fraction of pixels classified as one of six land covers |
| system_index | Feature identification from Google Earth Engine |
| id | Feature identification from Google Earth Engine |
| right | Polygon coordinate |
| top | Polygon coordinate |
| left | Polygon coordinate |
| bottom | Polygon coordinate |

**
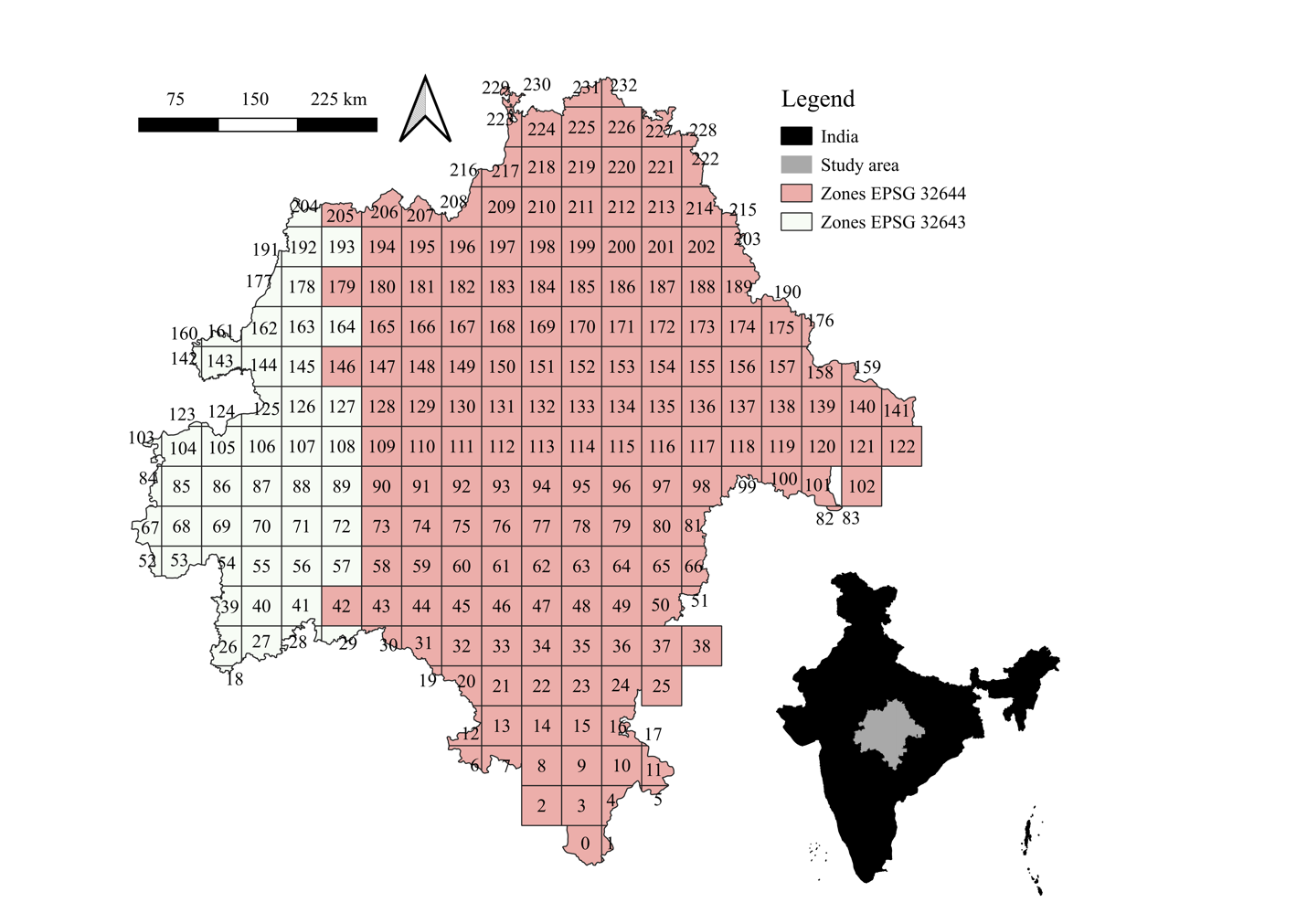
**

**Supplementary Figure 1.** Our study area in central India was broken into 233 study area zones. Each number refers to zone that has a land cover classification and BGI classification available for download. A shapefile of the study area zones is available to download. Land cover classification data is available with coordinate reference system (CRS) WGS 84 EPSG 32643 or 32644; zones in white are available with CRS WGS 84 EPSG 32643.

**
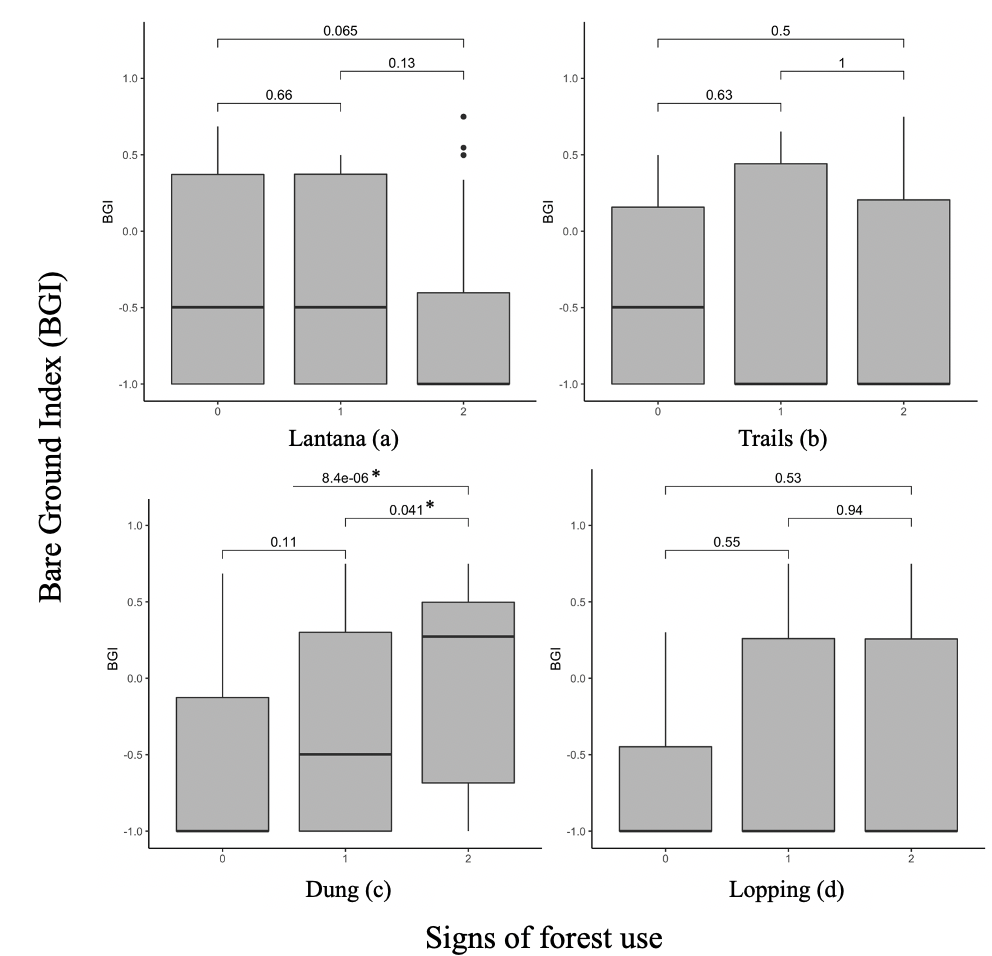
**

**Supplementary Figure 2.** The average values of the Bare Ground Index (BGI) in ground validation locations according to the level (0, 1 or 2) of signs of forest use. Validation locations were identified at the center of any given 90 meter pixel and signs of forest use within a 15 m radius of the point were recorded. Signs of forest use included lantana (a), trails (b), cattle dung (c), and tree lopping (d) and were categorized as 0 (no signs), 1 (1 or 2 signs), or 2 (3 or greater signs) for the same use type. Plots show the mean and the interquartile range (in gray) of the BGI. We compared the average BGI values of areas with different levels of forest use using a Wilcoxon rank sum test. * denotes a p < 0.05. There was a significant positive association between the presence of cattle dung and BGI.
